# Influence of Heating and Cyclic Tension on the Induction of Heat Shock Proteins and Bone-Related Proteins by MC3T3-E1 Cells

**DOI:** 10.1155/2014/354260

**Published:** 2014-06-11

**Authors:** Eunna Chung, Alana Cherrell Sampson, Marissa Nichole Rylander

**Affiliations:** ^1^Virginia Tech-Wake Forest University School of Biomedical Engineering and Sciences, Virginia Tech, ICTAS Building, Stanger Street (MC 0298), Blacksburg, VA 24061, USA; ^2^Department of Mechanical Engineering, Virginia Tech, ICTAS Building, Stanger Street (MC 0298), Blacksburg, VA 24061, USA

## Abstract

Stress conditioning (e.g., thermal, shear, and tensile stress) of bone cells has been shown to enhance healing. However, prior studies have not investigated whether combined stress could synergistically promote bone regeneration. This study explored the impact of combined thermal and tensile stress on the induction of heat shock proteins (HSPs) and bone-related proteins by a murine preosteoblast cell line (MC3T3-E1). Cells were exposed to thermal stress using a water bath (44°C for 4 or 8 minutes) with postheating incubation (37°C for 4 hours) followed by exposure to cyclic strain (equibiaxial 3%, 0.2 Hz, cycle of 10-second tensile stress followed by 10-second rest). Combined thermal stress and tensile stress induced mRNA expression of HSP27 (1.41 relative fold induction (RFI) compared to sham-treated control), HSP70 (5.55 RFI), and osteopontin (1.44 RFI) but suppressed matrix metalloproteinase-9 (0.6 RFI) compared to the control. Combined thermal and tensile stress increased vascular endothelial growth factor (VEGF) secretion into the culture supernatant (1.54-fold increase compared to the control). Therefore, combined thermal and mechanical stress preconditioning can enhance HSP induction and influence protein expression important for bone tissue healing.

## 1. Introduction

Bone is exposed to complex mechanical cues during motion, such as tension, compression, and fluid shear stress [1]. These mechanical forces modulate cell morphology, proliferation, migration, differentiation, and production of bone-related proteins in cells via complex signaling cascades [2]. As a result, mechanical cues regulate bone growth by maintaining a fine balance between the bone-forming activity of osteoblasts and the bone-resorbing activity of osteoclasts. To build bone, osteoblasts produce extracellular matrix proteins such as osteocalcin (OCN), osteonectin (ON), osteopontin (OPN), and the enzyme alkaline phosphatase (ALP) [3–5]. Conversely, osteoclasts secrete enzymes like matrix metalloproteinase-9 (MMP-9) to digest the bone matrix [6]. Osteoprotegerin (OPG), an antiosteoclastic protein, reduces bone degradation by inhibiting osteoclast function [7]. Overall, bone-related proteins are indicators of osteoblastic/osteoclastic activity and are essential for maintaining proper bone physiology.

Numerous conditioning protocols involving mechanical stress or heating have been applied* in vitro* to osteoblasts or osteogenic stem cells to promote the regenerative potential of bone. These conditioning treatments are envisioned to generate a stress regimen to enhance the protective and regenerative capacity of bone cells without causing cell death [8]. The degree of mechanical strain is dictated by parameters such as magnitude [9, 10], frequency [11], duration [12], cyclic number [13], and mode (e.g., continuous/intermittent [14] and uniaxial/equibiaxial [14, 15]). Cyclic strain imposed by the Flexcell tension system, a commercially available tensile bioreactor, can upregulate bone-related proteins such as types I and III collagen [15], osteopontin (OPN) [16, 17], osteocalcin (OCN) [16, 17], vascular endothelial growth factor (VEGF) [12, 17], bone morphogenetic protein-2 (BMP-2) [17], transforming growth factor beta 1 (TGF-**β**1) [18], osteoprotegerin (OPG) [10], and cyclooxygenase-2 (COX-2) [9] in bone cells. Varying tensile stress parameters determine the magnitude of stress that cells experience and can therefore influence cell behavior.

Thermal stress can activate various intracellular mechanisms and cellular responses depending on the type of heating system (e.g., water bath, incubator [19], or laser irradiation [12]), loading temperature [20–22], and heating duration [22]. Numerous research groups agree that heating with temperatures of 46–50°C for less than 10 minutes or temperatures of 43-44°C for longer durations than 15 minutes may cause cytotoxicity or decreased protein production [22–25]. With mild thermal stress endothelial cells exhibit enhanced angiogenic capacity 24 hours following exposure to 41°C for 1 hour [8]. Direct application of thermal stress to bone [19] and indirect thermal stress conditioning by adding supernatant collected from heat-treated osteoblasts [26] can promote cell proliferation and upregulate OCN. Based on our previously published work, water bath heating at 44°C for 8 minutes induced heat shock proteins (HSPs) and bone-specific proteins, such as OPN [27]. Although thermal stress is known to modulate protein production, the mechanism by which stress modulates cell behavior is unknown.

Thermal and mechanical stress can elicit the cytoprotective effects of molecular chaperones known as heat shock proteins (HSPs) [28]. As multifunctional proteins, HSPs are involved in mitosis [29], differentiation [30, 31], cytoskeleton stabilization [32], intracellular processing of matrix proteins (e.g., collagen) [33], immune system control [34], and the wound healing process [35, 36]. HSPs are characterized according to their molecular weight (e.g. HSP27) and each HSP has distinct functions and expression profiles depending on external stresses and cell type. HSP47 is associated with the collagen synthesis process by binding to procollagen [33, 37]. Both HSP27 and HSP70 rescue stressed cells from apoptotic cell death through various mechanisms [30]. Therapeutic approaches using the beneficial aspects of HSPs have been investigated in sepsis, transplantation, skin damage, and ischemic diseases of bone, brain, and heart, as reviewed by Jäättelä [38]. HSP27, HSP47, and HSP70 are highly expressed in bone-forming osteoblasts of rat bone demonstrated by immunohistochemistry [39]. In addition, heating using an incubator and water bath at 42–50°C can upregulate HSP expression [19, 22, 28, 40]. HSP70 expression can increase in trabecular meshwork cells and tendon fibroblasts following cyclic tension [41, 42]. Our previous study demonstrated heating and tension alone induced gene expression for all previously mentioned HSPs [27, 43].

Taken together, there may be a critical correlation between HSP expression and protective/osteogenic responses of bone cells in response to stress. Prior studies utilizing mechanical [10, 12, 15, 16, 44, 45] and thermal [19, 46] stress conditioning have suggested that these stresses can be beneficial stimulators for bone cell activity. However, few studies have explored the potential of combined stress protocols to improve bone regeneration. This study investigated whether the combination of thermal and mechanical stress could facilitate enhancement in cell proliferation, induction of HSPs, and upregulation of angiogenic/osteogenic proteins. Preosteoblasts were exposed to a single dose of water bath heating (44°C, 4 or 8 minutes) and cyclic strain (equibiaxial 3% elongation, 0.2 Hz, cycle of 10-second tension followed by a 10-second resting phase), and subsequently we evaluated cell morphology, cell proliferation, induction of HSPs (HSP27, HSP47, and HSP70), osteogenic matrix proteins (e.g., collagens, OPN, and OCN), and enzyme levels of MMP-9. An angiogenic growth factor, VEGF, and an antiosteoclastic cytokine, OPG, served as additional metrics for evaluating the contribution of stress conditioning to osteogenesis. To the best of our knowledge, this is the first study exploring the effects of combined thermal and mechanical stress on preosteoblasts. The results from our study may provide a better understanding of cellular response to multiple stresses and may also be useful in developing a stress protocol that stimulates cell activity for bone regeneration.

## 2. Materials and Methods

### 2.1. Cell Culture and Preparation for Stress Treatment

A murine preosteoblastic cell line, MC3T3-E1 (subclone 4, American Type Culture Collection, Manassas, VA), was cultured with growth media composed of alpha minimal essential medium (**α**MEM) (Mediatech, Manassas, VA) supplemented with 10% fetal bovine serum (FBS) and 1% penicillin-streptomycin (PS) in a 5% CO_2_ incubator at 37°C. Prior to stress conditioning, cells were plated in a 6-well BioFlex plate at 2 × 10^5^ cells per well and cultured overnight (for 16-17 hours) to allow cell adhesion.

### 2.2. Combined Stress Conditioning with Heating and Cyclic Tension

**Figure 1:**
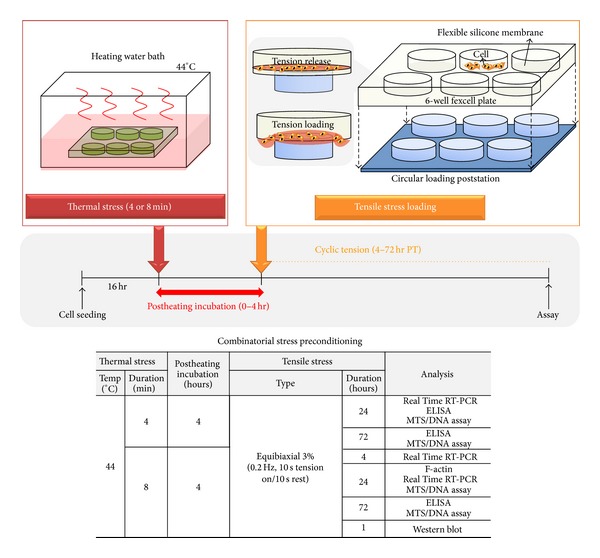
Method of combined thermal and tensile stress conditioning for MC3T3-E1 monolayers. Cells were seeded on 6-well BioFlex plates with flexible culture substrate 16 hours before stress preconditioning. First, thermal stress was applied by heating in a water bath at 44°C for 4 or 8 minutes followed by 4-hour postheating incubation at 37°C and cyclic tensile stress conditioning protocols of equibiaxial 3% maximum elongation and 0.2 Hz (cycle of 10-second tension followed by a 10-second resting phase) using the Flexcell tension system. PT denotes the period of tensile stress treatment before cell analysis. The table summarizes the specific preconditioning protocols and associated assays performed.

After cell adhesion, cells underwent the following stress conditioning protocols consisting of four different test groups: (1) sham-treated control, (2) thermal stress only, (3) tensile stress only, and (4) combined thermal and tensile stress. During thermal stress treatments, heating media, which consisted of Eagle's MEM without L-glutamine (Sigma-Aldrich), were added to fill the entire volume of the wells within the Flexcell plate. This specialized media composition was used because, at high temperatures experienced with thermal stress, L-glutamine can degrade quickly and become cytotoxic to cells. To maintain consistency, these media were used during all stress treatments. Thermal stress was applied by submerging the Flexcell well plate in a water bath (ISOTEMP 210, Fisher Scientific) set at 44°C for heating durations of 4 or 8 minutes, similar to prior work by Rylander et al. [22, 47]. For samples exposed to tension only, Flexcell tension plus system (Flexcell International Corporation, Hillsborough, NC) was utilized to apply cyclic tensile stress conditioning protocols of equibiaxial 3% maximum elongation and 0.2 Hz (cycle of 10-second tension followed by a 10-second resting phase) for identical tension durations used during combined stress treatments. A circular loading post (diameter = 25 mm) was used to apply equibiaxial tension. Combinatorial stress treatment was conducted as depicted in Figure 1 using identical methods for thermal and tensile conditioning described previously. For these experiments, a single dose of water bath heating (44°C, 4 or 8 minutes) was applied followed by 4-hour postheating incubation at 37°C. Subsequently, cells were exposed to cyclic tension using the Flexcell tension system for varying durations (1–72 hours period of tensile stress (PT)) depending on test measurements. PT time denotes the duration of tensile stress conditioning and the timepoint for collecting data. Sham-treated control groups were not exposed to any stress treatment but were cultivated with identical media as stress-treated groups and maintained in an incubator. Each type of measurement within a single test group was performed at the same time regardless of whether tension or heating was applied. For poststress recovery, osteogenic media (**α**MEM supplemented with 50 *μ*g/mL L-ascorbic acid, 10 mM**β**-glycerol phosphate, 10% FBS, and 1% PS) was added to cells and all samples were returned to a 5% CO_2_ incubator at 37°C. Media formulation was based on a previously described protocol for osteogenic media which was demonstrated to be conducive for differentiation of MC3T3-E1 cells [48].

### 2.3. Morphology Analysis

Cell morphology following stress treatment was visualized by fixing the cells immediately after stress and staining for F-actin, a cellular skeleton protein, using rhodamine phalloidin (Invitrogen). Cells were fixed with 3.7% paraformaldehyde in a phosphate buffered solution (PBS) (Fisher Scientific) and permeabilized using 0.1% Triton X-100 (Sigma)/PBS. For blocking, samples were incubated in 1% bovine serum albumin (Amersham) dissolved in PBS for 30 minutes at room temperature followed by 20-minute incubation in rhodamine phalloidin solution in the dark. For nucleus counterstaining, cells were mounted with VECTASHIELD mounting medium with DAPI (DAPI: 4′,6-diamidino-2-phenylindole) (Vector Laboratories). Stained images were acquired using a fluorescent inverted microscope (CTR6500, Leica Microsystems).

### 2.4. Proliferation Assay

Cell proliferation was measured at 24 and 72 hours following thermal (44°C, 4 and 8 minutes) and tensile stress applied independently or in combination. We implemented two different assays: 3-(4,5-dimethylthiazol-2-yl)-5-(3-carboxymethoxyphenyl)-2-(4-sulfophenyl)-2H-tetrazolium (MTS) assay using CellTiter96 Aqueous one solution cell proliferation assay (Promega Corporation, Madison, WI) and DNA assay using Quant-iT PicoGreen dsDNA reagent kit (Invitrogen) according to the manufacturer's protocols. MTS stock solution was mixed with basal  **α**MEM without FBS and PS (the volume ratio of MTS stock to media was 1 : 5). Diluted MTS working solution was added to cultured cells. After 4-hour incubation at 37°C, the solution was transferred to a 96-well plate and optical density was measured at 490 nm by a microplate reader (SpectraMax M2^e^, Molecular Devices, Sunnyvale, CA). DNA was isolated at identical timepoints as the MTS assay. In brief, cells were lysed using Tris-EDTA buffer (10 mM Tris, 1 mM EDTA, pH 8.0, Fisher Scientific) including 0.1% Triton X-100 (Sigma) and 0.1 mg/mL proteinase K (Fisher Scientific). Cell lysate was incubated at 56°C overnight and transferred into a 96-well plate with standard solutions. Quant-iT PicoGreen dsDNA was added to each sample at a volume ratio of 1 : 1 and incubated at room temperature in the dark for 3 minutes. Fluorescence of each sample was measured by a microplate reader (SpectraMax M2^e^, Molecular Devices, Sunnyvale, CA) set at the 480/520 nm (excitation/emission).

### 2.5. Quantitative Real Time RT-PCR

Gene expression of HSPs and bone-related proteins was measured following individual or combined treatment with thermal and tensile stress. RNA was isolated by spin protocol using an RNeasy Mini kit (Qiagen) and a QIAshredder (Qiagen), according to the manufacturer's protocol. RNA isolation was performed immediately after 4–72-hour cyclic tension or directly following heating at 44°C and 4-hour postheating incubation at 37°C. Isolated RNA was converted to cDNA using reverse transcription system (Promega). RNA from each sample was reacted at 25°C for 10 minutes and 42°C for 45 minutes followed by heating at 99°C for 5 minutes. After reverse transcription, cDNA samples were mixed with Taqman PCR Master Mix (Applied Biosystems) and each specific primer and polymerized in a 7300 Real Time PCR System (Applied Biosystems). The PCR reaction was performed at 50°C for 2 minutes followed by 95°C for 10 minutes. For each polymerization (total PCR reaction = 45 cycles), temperature was set at 95°C for 15 seconds and 60°C for 1 minute. Taqman gene expression assay (Applied Biosystems) for specific gene detection was used as a primer and probe as follows: GAPDH (Mm99999915_g1), HSP27 (Mm00517908_m1), HSP47 (Mm00438056_m1), HSP70 (Mm03038954_s1), OPN (Mm01611440_mH), OPG (Mm01205928_m1), MMP-9 (Mm00600164_g1), ALP (liver/bone/kidney) (Mm01187113_g1), OCN (Mm00649782_gH), type I collagen (alpha 1) (Mm00801666_g1), and VEGF (Mm00437308_m1). Relative fold induction (RFI) of each mRNA was calculated according to the 2^−ΔΔC_T_^ method used in Lee et al.'s study [49]. Threshold cycle (C_T_), derived using SDS v1.2× system software of 7300 Real Time PCR System, denotes the fractional cycle number at threshold polymerized gene and ΔΔC_T_ was derived from the following equation: (C_T_ of target gene − C_T_ of GAPDH)_treated  group_ − (C_T_ of target gene − C_T_ of GAPDH)_control  group_ [49]. Treated groups denote thermal stress alone, tension alone, or combined thermal and tensile stress treatments. Control groups indicate sham-treated cells without heating and tension.

### 2.6. Enzyme-Linked Immunosorbent Assay Analysis (ELISA)

Protein secretion by MC3T3-E1 cells following thermal and tensile stress independently or in combination was analyzed by enzyme-linked immunosorbent assay (ELISA), according to the manufacturer's protocol. In brief, immediately after 24-hour (for VEGF) and 72-hour (for OPN, OPG, and MMP-9) cyclic tension loading, the conditioned osteogenic culture supernatant was collected. For untreated and heated samples, supernatant was isolated at identical timepoints although no tension was applied. The concentrations of OPG, VEGF, OPN, and MMP-9 in the conditioned cell culture medium were determined using Quantikine ELISA (R&D Systems). The culture supernatant was added to a 96-well microplate coated with antibodies for the desired proteins and incubated for 2 hours at room temperature. After washing, samples were incubated in the conjugate for 2 hours. Subsequently, peroxidase substrate solution was added to initiate an enzymatic reaction that generates a colored product in proportion to protein concentrations in each sample. After 30 minutes, the optical absorbance was measured at 450 nm by a microplate reader (SpectraMax M2^e^, Molecular Devices, Sunnyvale, CA) and converted into the concentration level using a standard absorbance curve.

### 2.7. Statistical Analysis

All data and graphs are presented as mean ± standard deviation. Experimental groups with a minimum of three replicates were tested and analyzed independently. Using JMP 8.0 statistical software, a one-way ANOVA and a Tukey multiple comparison test were performed to compare the means between each group. The significance of each treatment in the study was defined by a *P* value lower than 0.05.

## 3. Results and Discussion

This study investigated the effect of combined tensile and thermal stress on preosteoblasts by evaluating their* in vitro* osteogenic response. Due to the varied response of cells to diverse stress conditions, the parameters used in our stress protocols were selected based on studies in the literature. Our heating protocols were chosen based upon our previous published work in which heating at 44°C for periods less than 10 minutes induced HSPs rapidly without any cytotoxicity [27]. Our heating protocols are also comparable to those used by other groups at temperatures of 40–43°C (for heating durations of 30 minutes to 1 hour) [26, 28, 50]. Although the water bath was set to ca. 44°C, the cell culture vessels required time to equilibrate to the surrounding water bath temperature (data not shown here) causing the cells to experience temperatures in the range of 40–43°C for short periods. The tensile stress protocol used in our study was selected based on prior literature, which employed tensile stress conditioning typically lower than 18% strain and documented positive osteogenic effects inducing upregulation of collagen, VEGF, and COX-2 [9, 15, 51, 52]. Furthermore, 0.1–1 Hz frequency and 6 cycle numbers per minute also have been investigated commonly in bone-related studies [10, 15, 51, 53]. In Winter et al.'s study, intermittent stretching induced higher levels of DNA and calcium in osteoblasts compared to continuous tensile stress [14], suggesting the importance of rest periods to enhance cellular response to tension. Also, equibiaxial strain has been demonstrated to increase collagen expression, cell proliferation, and VEGF in bone-related cells [9, 15, 52]. Based on these studies, our tensile stress protocols employed 3% cyclic equibiaxial stretching with 0.2 Hz frequency and 6 cycle numbers per minute in an intermittent manner (10-second tension followed by 10-second rest period) for MC3T3-E1 preosteoblasts. To improve the beneficial effects seen with individual thermal and tensile stress, this study investigated the ability of combined stress protocols to enhance bone development.

### 3.1. Effect of Combined Heating and Cyclic Tension on Cell Morphology

**Figure 2:**
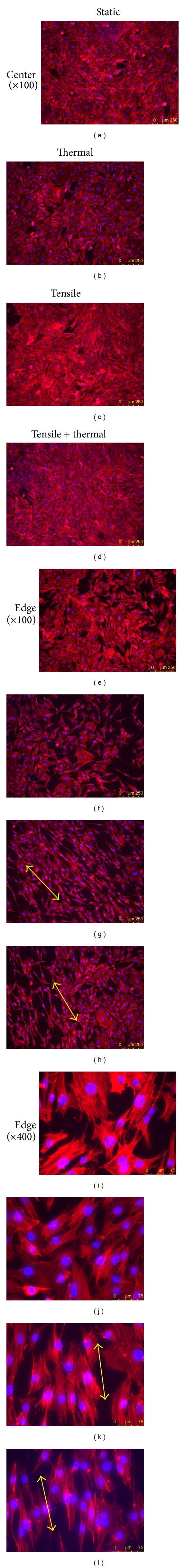
Cell morphology as visualized by F-actin staining following stress conditioning. Cell morphology is shown in response to thermal stress ((b), (f), and (j)), tensile stress ((c), (g), and (k)), and combined thermal and tensile stress ((d, (h), and (l)). Static-cultured cells ((a), (e), and (i)) were used as a control. Varying positions within the well were imaged, including the center ((a)–(d)) and the edge under 100x ((e)–(h)) and 400x magnification ((i)–(l)). Yellow arrows denote the direction of tensile stress generated due to the loading post. Heating at 44°C for 8 minutes (postheating incubation = 4 hours); cyclic tension (equibiaxial 3%, 0.2 Hz, 10-second tension on/10-second rest, 24 hours).

Cell morphology was visualized by F-actin fluorescence staining to determine the effect of heating and mechanical tensile stress alone or in combination (Figure 2). Similar to sham-treated controls, cells had a broad, flat morphology and there was no apparent alteration in response to heating. However, tension and combined stress caused cell alignment and elongation around the perimeter of the culture plate. Mechanical stress can influence cell morphology by disrupting cellular focal adhesions that connect the cytoskeleton to the substrate. The subsequent rearrangement of the actin cytoskeleton can cause cells to align in the direction of stretching (yellow arrows in Figure 2).

### 3.2. Effect of Combined Heating and Cyclic Tension on Cell Proliferation

**Figure 3:**
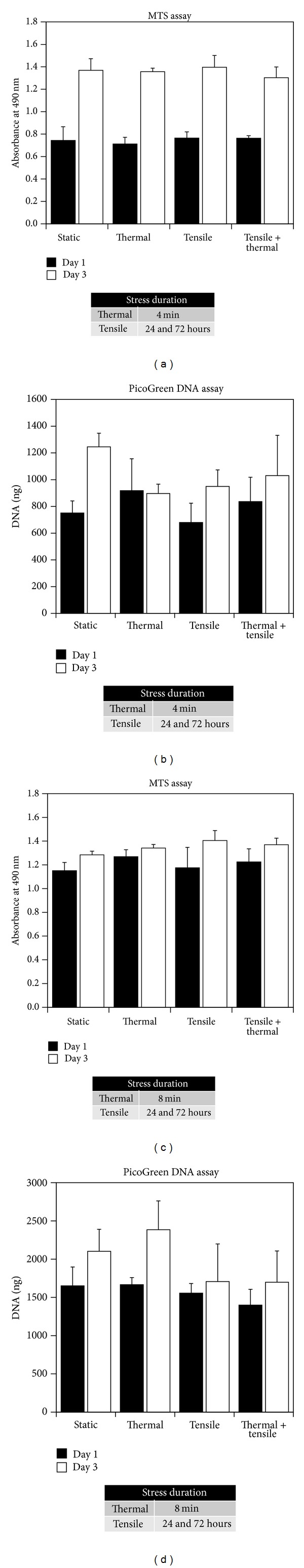
MC3T3-E1 proliferation was measured 24 and 72 hours following a single dose of thermal stress (44°C, 4 or 8 minutes, postheating incubation = 4 hours) and cyclic tension (equibiaxial 3%, 0.2 Hz, 10-second tension on/10-second rest, 24 and 72 hours) individually or in combination. Cell proliferation was measured using MTS and PicoGreen DNA assay with varying heating durations of 4 minutes ((a) and (b)) and 8 minutes ((c) and (d)) (*n* = 3).

We investigated MC3T3-E1 proliferation by measuring metabolic activity and DNA concentrations using an MTS assay and Quant-iT PicoGreen dsDNA assay, respectively (Figure 3). Cells did not show any associated cytotoxicity following heating for individual and combinatorial conditioning of 4 (Figures 3(a) and 3(b)) or 8 minutes (Figures 3(c) and 3(d)) of heating and tension (24 and 72 hours). In addition, cells did not experience apoptotic damage following stress as evidenced by minimal changes in metabolic activity and DNA concentrations for longer cultivation (72-hour PT) in all groups. There were not significant differences in cell metabolic activities depending on stress types.

Previous studies investigating cell proliferation or cytotoxicity in response to stress have shown outcomes comparable to our results when similar stress conditions were used. For thermal stress, Riederer et al. showed that low-level heat treatment at 42°C for 1 hour using an incubator did not significantly influence the proliferation rate of human myoblasts [50]. Another study described that water bath heating at 39–41°C for 1 hour induced a slight, but nonsignificant, increase in cell proliferation [19]. The effect of tensile stress on proliferation has also been evaluated in prior studies using similar stress conditions as our work. For example, Huang et al. demonstrated tension (3%, 0.1 Hz) slightly increased metabolic activity of MSCs on day 1 but exhibited similar levels as static-cultured cells on days 3 and 5 [53]. The results from these studies are comparable to our MTS and DNA data, which shows no significant increases in cell proliferation following stress treatment on MC3T3-E1 cells. Furthermore, our study did not demonstrate any statistically significant reduction in cell proliferation, suggesting that our stress conditioning protocols do not induce apoptosis or negatively affect metabolic activity of the cells.

### 3.3. Effect of Combined Heating and Cyclic Tension on HSP Expression

**Figure 4:**
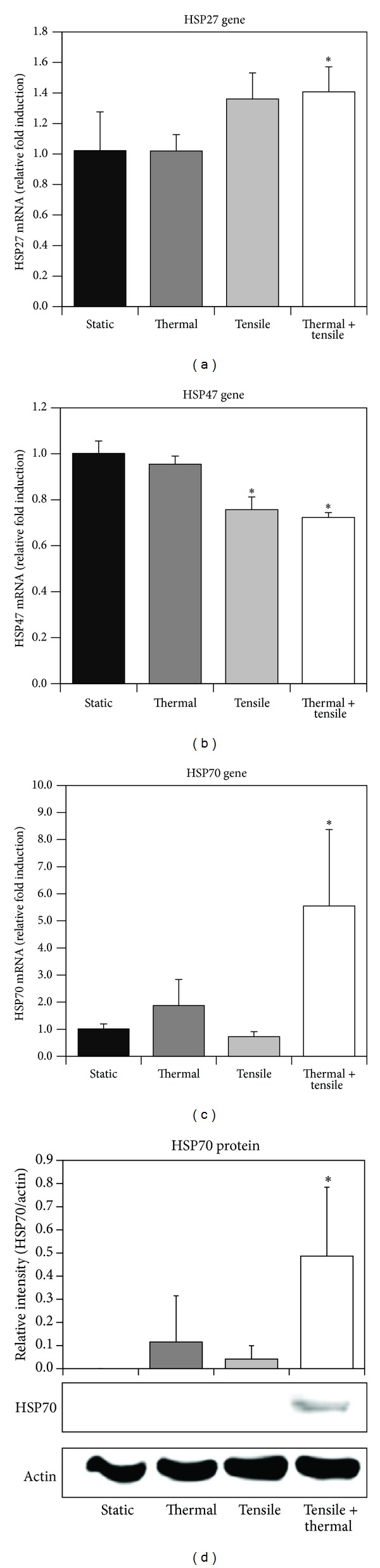
HSP (HSP27, HSP47, and HSP70) expression following a single dose of thermal stress (44°C, 4 or 8 minutes) and cyclic tension (equibiaxial 3%, 0.2 Hz, 10-second tension on/10-second rest) individually or in combination. Gene expression of HSP27 (a), HSP47 (b), and HSP70 (c) following heating for 8 minutes and 4-hour cyclic strain (*n* = 4 for (a)–(c)). Protein expression of HSP70 following heating for 4 minutes and 1-hour cyclic tension (d). ∗ denotes statistical significance between stress-treated and sham-treated control groups (*P* < 0.05) (*n* = 3).

To evaluate the influence of stress conditioning on cytoprotective proteins and the cellular stress response, we measured gene expression and protein secretion of HSPs following individual and combined heating (44°C, 4 or 8 minutes) and cyclic mechanical strain (24 hours, 4 hours, or 1 hour) conditioning. Messenger RNA (mRNA) expression for HSPs (HSP27, HSP47, and HSP70) and the protein level of HSP70 after stress treatments are shown in Figure 4. Long durations of cyclic tension (24 hours) alone or in combination with 4- or 8-minute heating showed no significant induction of mRNA for any of the HSPs measured (data not shown). For shorter durations of tensile conditioning (4 hours) and 8-minute heating, individual heating and cyclic tension did not produce a substantial effect, but combined stress treatments significantly altered HSP expression. Combined stress conditioning of 8-minute heating followed by 4 hours of cyclic tension caused significant induction of HSP70 (5.55 RFI) and HSP27 (1.41 RFI) mRNA compared to tensile or thermal stress alone (Figures 4(a) and 4(c)). In addition, HSP47 mRNA was suppressed by tension alone (0.76 RFI), but combined stress caused a reduction in HSP47 mRNA expression (0.72 RFI) (Figure 4(b)). Furthermore, protein induction of HSP70 following combined heating (at 44°C for 4 minutes) and 1-hour tension (Figure 4(d)) was significantly promoted.

Our data suggests that combined stress conditioning has a greater influence on HSP expression than individual stress. In addition, the degree of HSP induction in response to the type of stress varies among different HSPs. For example, we observed that HSP70 gene (5.55 RFI) and protein expression were more sensitive to combined stress treatments compared to HSP27 (1.41 RFI) and HSP47 (0.72 RFI). Also, we observed that tensile stress alone significantly altered HSP47 mRNA but had little influence on HSP27. These results are similar to findings in prior studies which have shown differential HSP induction depending on the type and degree of stress [27, 43]. Although cellular induction of HSPs varies depending on the stimuli, the ability of our combined tensile and thermal stress protocol to induce significant changes in gene expression for all HSPs measured compared to the control suggests that combined stress can provide the appropriate level of stimulation to broadly influence cellular protein production.

In our study, the upregulation of HSP70 and HSP27 following combined stress has important implications since previous studies have shown the cytoprotective effects of HSPs in a variety of tissues. For example, elevated levels of HSP70 in the heart following stress preconditioning resulted in increased tolerance of myocardiocytes to subsequent ischemia [54]. Zheng et al. demonstrated that overexpression of the HSP70 gene in mice prevented cell death after brain injury [55]. Prior studies have also revealed that the level of HSP induction is directly related to the amount of protection [56]. Based on this, our data suggests that combined stress conditioning has a greater ability to invoke cytoprotection by upregulating HSPs, compared to individual stress. Despite exposing cells to a higher degree of stress, the minimal changes in cellular metabolism and cell proliferation we observed demonstrate that our combined stress protocol can modify cellular response without causing cytotoxic effects. Although the mechanism is not known, combined stress can have a significant impact by exposing cells to multiple stresses that can act individually or synergistically to enhance cellular response.

Our data showing induction of HSP70 expression in response to heating is consistent with prior studies showing elevated HSP70 in chondrocytes [57] and endothelial cells [22] in response to heating. Shui and Scutt demonstrated that thermal stress (ca. 41–45°C for 1 hour or 39°C for 96 hours) conditioning of bone cells induced beneficial osteogenic effects by significantly enhancing calcium production, ALP activity, and HSP70 following heating at 39°C for 96 hours [19]. Our prior work [27] showed significant induction of mRNA of all HSPs at 8-hour postheating following 8-minute heating, but HSP upregulation by heating in this study was lower or did not exhibit induction. This may be due to differences in cell density and culture plates used (general plastic T-flask for previous study and Flexcell BioFlex plate coated with type I collagen for the current study). Furthermore, in terms of tensile stress, our previous studies showed 1% tension transiently induced HSP27 (1.82 RFI) and HSP70 (1.53 RFI) mRNA at day 3, but this induction level was relatively lower than induction by heating [27, 43]. To our knowledge this is the first study measuring HSP70 induction in preosteoblasts in response to combined heating and tension.

### 3.4. Effect of Combined Heating and Cyclic Tension on Bone-Related Proteins

**Figure 5:**
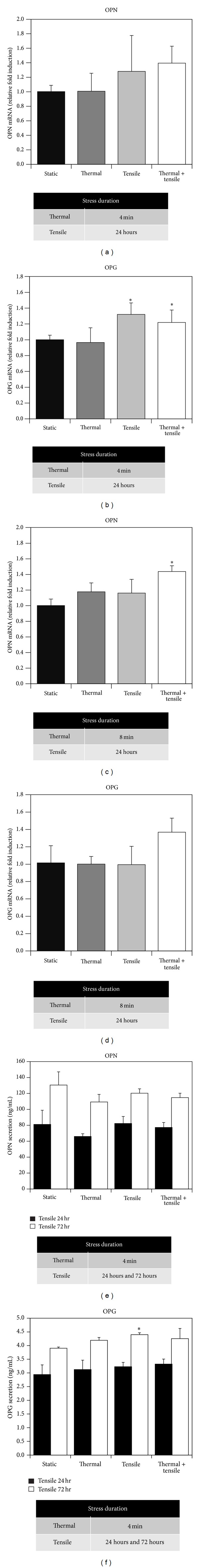
Gene expression and protein secretion of OPN and OPG by MC3T3-E1 cells following a single dose of thermal stress (44°C, 4 or 8 minutes) and cyclic tension (equibiaxial 3%, 0.2 Hz, 10-second tension on/10-second rest, 24 hours or 72 hours) individually or in combination. OPN and OPG gene expression is shown in response to varying heating durations of 4 minutes ((a) and (b)) and 8 minutes ((c) and (d)) alone or in combination with cyclic tension for 24 hours. OPN (e) and OPG (f) concentration secreted in the culture supernatant (measured by ELISA) is shown following heating for 4 minutes and cyclic tension for 24 or 72 hours. ∗ denotes statistical significance between stress-treated and sham-treated groups (*P* < 0.05) (*n* = 4).

Relative changes in gene expression and protein secretion of bone-related proteins in response to heat (44°C, 4 and 8 minutes) and/or tensile stress (24 hours) are shown in Figure 5. OPN mRNA was slightly increased by the combination of heating for 4 and 8 minutes and 24 hours of tension compared to either stress individually (Figures 5(a) and 5(c)). OPG mRNA increased following 24-hour tension alone and in combination with 4-minute heating (Figure 5(b)). Although tension alone exhibited the greatest upregulation of OPG, combined stress stimulated comparable mRNA levels. OCN mRNA was not affected by any of the stress protocols (data not shown). Secreted OPN and OPG in the culture media were analyzed by ELISA kits following 4-minute heating and 24- or 72-hour tension (Figures 5(e) and 5(f)). There was a statistical difference between OPG induction (13% increase) following tension for 72 hours compared to the control. However, OPN secretion did not show any significant difference between test groups at 24- and 72-hour PT.

The upregulation of OPG, a protein that inhibits osteoclast differentiation, and OPN, a protein that mediates cell-bone ECM interactions, can be beneficial for bone growth by limiting osteoclast activity, decreasing bone resorption, and regulating ECM maturation. In this study, combined stress was influential in increasing gene expression of OPG and OPN. Previous studies have shown comparable results to our data. For example, in Tang et al.'s study, OPG mRNA in MC3T3-E1 cells was increased in a magnitude-dependent manner following 6–18% (6 cycles per minute) for 24 hours but the relative fold induction of 6% stretching to sham-treated cells was lower than 2 RFI [10]. Similar to our previous study [43], OCN and OPN genes appear not to be influenced by 24-hour tension alone. However, heating (for 8 minutes) alone induced mRNA expression of OCN (3.8 RFI), OPN (1.8 RFI), and OPG (2.1 RFI) genes at 8-hour postheating in our previous study [27]. Furthermore, in our prior work [43], we observed significant OPG secretion in response to 5% tensile stress preconditioning, comparable to the current study employing 3% tension, suggesting that OPG can be upregulated by tension, but not significantly by thermal stress. The discrepancy may be caused by culturing cells on a type I collagen-coated flexible substrate and the use of a different FBS concentration in the osteogenic culture media between these two studies. In addition, our study analyzed type I collagen, which showed no apparent changes in gene expression regardless of which stress conditioning protocol was applied (data not shown). These results are comparable to previous studies [27], where type I collagen mRNA was not influenced by either heating (for 4 or 8 minutes) and tension (for 24 hours) or mechanical strain of 10–12% magnitude (0.1 Hz, cycle number 6 per minute for 24 hours) [51]. Based on these findings, type I collagen gene expression may not be responsive to short-term stress conditioning. However, even without drastic changes in gene expression, collagen deposition can still be influenced by stress and further investigation is necessary.

### 3.5. Effect of Combined Heating and Cyclic Tension on MMP-9 Expression

**Figure 6:**
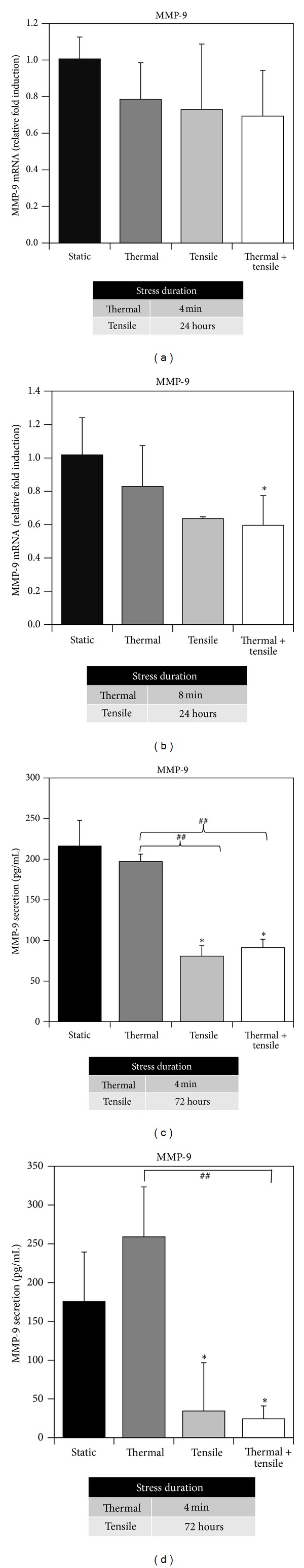
MMP-9 gene expression and protein secretion by MC3T3-E1 cells following a single dose of thermal stress (44°C, 4 or 8 minutes) and cyclic tension (equibiaxial 3%, 0.2 Hz, 10-second tension on/10-second rest) alone or in combination. MMP-9 mRNA expression was measured with RT-PCR following heating for 4 minutes (a) or 8 minutes (b) alone or in combination with cyclic tension for 24 hours. MMP-9 protein secretion was measured with ELISA in response to heating for 4 minutes (c) or 8 minutes (d) alone or in combination with cyclic tension for 72 hours. ∗ denotes statistical significance between stress-treated and sham-treated control groups (*P* < 0.05). ## denotes statistical significance between individual thermal stress and combined thermal and tensile stress (*n* = 8 for (a); *n* = 4 for (b)–(d)).

MMP-9 mRNA expression and protein secretion were suppressed in response to thermal (4 or 8 minutes) and tensile stress (24 or 72 hours) alone or in combination (Figure 6). Combined heating and tensile stress caused MMP-9 mRNA expression and protein secretion to decrease at 24-hour PT and 72-hour PT, respectively (Figures 6(a)–6(d)). Although MMP-9 secretion at 72-hour PT increased with 8-minute heating alone (Figure 6(d)), the greatest change in secretion was observed with combined stress and individual tensile stress. Combined 8 minute heating and tension was able to significantly decrease gene expression of MMP-9 compared to the control.

Similar to our previous study, MMP-9 secretion was inhibited by heating [27] and 3% mechanical strain [43]. Therefore, MMP-9 mRNA in MC3T3-E1 cells appears to be suppressed by cyclic tensile stress following short periods of tension. Our results compare well with a prior study using a Flexcell bioreactor applying 10% tension (0.5 Hz) which caused diminished MMP-9 gene expression of RAW264.7 osteoclastic cells [58]. Based on these studies, our results imply that tension is the main factor stimulating MMP-9 suppression. Since combined stress yields similar or more pronounced levels of MMP-9 inhibition, we can deduce that tension contributes significantly to the results observed with combined stress. Although the mechanism is unknown, combined stress could promote enhanced effects over individual stress by exposing cells to multiple stimuli that can act individually or synergistically to promote an enhanced cellular response.

MC3T3-E1 cells express several types of MMPs including MMP-2, MMP-9, and MMP-13 [59], but MMP-9 was chosen because it is a well-known enzyme involved in bone remodeling/development. Although primarily associated with osteoclasts, this enzyme is also produced by osteoblasts to influence osteoclast activity [58, 60, 61]. MMP-9 overexpression in the bone microenvironment could be an osteoclastic activator for bone resorption. Recently, reducing MMP-9 expression has become a promising therapeutic strategy for bone diseases with high osteoclast activity such as osteoporosis. Therefore, MMP-9 suppression by stress conditioning could provide a beneficial impact for bone regeneration. Despite the suggested MMP-9 suppression by tension alone or in combination with heating, this phenomenon should be investigated further to determine whether suppressed MMP-9 can alter bone development.

### 3.6. Effect of Combined Heating and Cyclic Tension on VEGF Expression

**Figure 7:**
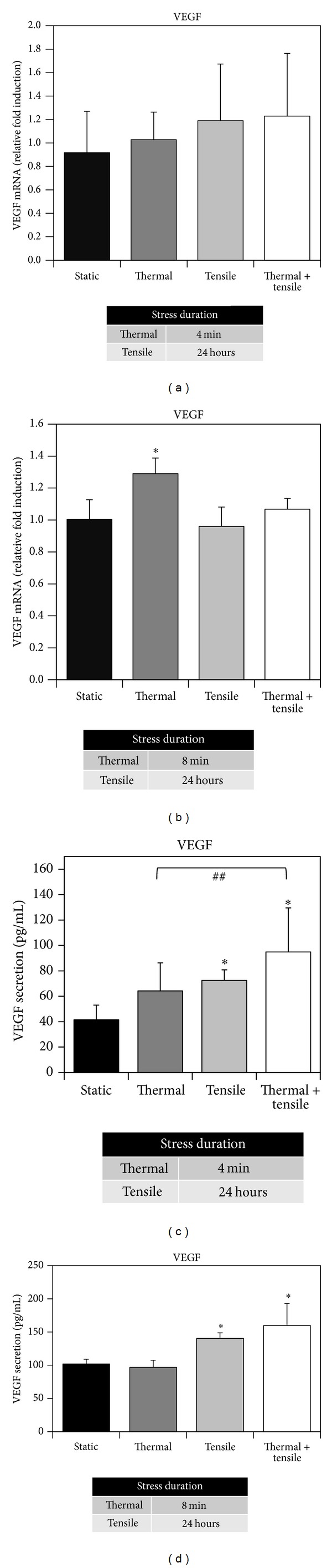
VEGF gene and protein expression by MC3T3-E1 cells following a single dose of thermal stress (44°C, 4 or 8 minutes) and cyclic tension for 24 hours (equibiaxial 3%, 0.2 Hz, 10-second tension on/10-second rest) alone or in combination. VEGF mRNA expression was measured with RT-PCR following heating for 4 minutes (a) or 8 minutes (b) alone or in combination with cyclic tension. VEGF protein secretion was measured with ELISA in response to heating for 4 minutes (c) or 8 minutes (d) alone or in combination with cyclic tension. ∗ denotes statistical significance between stress-treated and sham-treated control groups (*P* < 0.05). ## denotes statistical significance between individual thermal stress and combined thermal and tensile stress (*n* = 8 for (a) and (c); *n* = 4 for (b) and (d)).

VEGF mRNA and protein secretion were measured following individual and combined thermal (44°C, 4 and 8 minutes) and tensile stress (Figure 7). Stress conditioning did not influence VEGF mRNA expression (Figures 7(a) and 7(b)), except for 8-minute heating alone (Figure 7(b)). VEGF secretion significantly increased with tension alone, but slightly higher concentrations were observed after combined heating and tensile stress compared to tension only, as demonstrated by ELISA data (Figures 7(c) and 7(d)): 4-minute heating and tension (Figure 7(c)) (static 41.5 pg/mL; thermal 64.2; tensile 72.5; combined 94.9); 8 minute heating and tension tension (Figure 7(d)) (102.0; 95.5; 138.9; 156.6).

Prior studies have suggested that thermal or mechanical stress can stimulate VEGF induction [52, 62, 63]. For example, heating at 42°C for 15 minutes using a heating blanket has been shown to induce VEGF in rat cardiac tissue at 4–72-hour postheating [62]. Kim et al. showed an increase in VEGF expression after 90-minute heat stress at 43°C using a heating pad and infrared radiation at 42°C [63]. Tensile stress has been shown to rapidly promote VEGF gene expression in osteoblasts in response to 3-hour equibiaxial tension (10% magnitude) [52] and prior studies by our group have shown that tension can increase VEGF secretion [44]. These trends are comparable to results from our study, although VEGF gene expression and protein secretion were regulated differently depending on the type of stress. For example, our results show individual thermal stress upregulated VEGF gene expression but had no effect on protein secretion. For individual tensile stress, negligible effects in VEGF gene expression were observed, but these treatments were able to stimulate cells to secrete increased concentrations of VEGF. In addition, our results for combined stress followed a similar trend as individual tensile stress but exhibited slightly increased VEGF secretion levels, suggesting that tensile stress may be the dominant stimulus in our combined stress protocol. Although only a slight difference was observed, the increased VEGF secretion invoked by combined stress compared to tension alone/thermal alone suggests that exposing cells to multiple stresses may have an enhanced effect over individual stress. However, additional research is necessary to determine whether combined stress can induce more pronounced differences in VEGF secretion.

In our previous studies [27] VEGF gene expression was induced at 8-hour postheating by thermal stress (44°C, 8 minutes) and more significantly with growth factors (GFs) (i.e., BMP-2 and TGF**β**-1). In our prior study [43], tension alone did not cause VEGF gene induction, but the combination of tension and growth factors increased VEGF gene and protein upregulation compared to growth factor addition or tension alone. Although GFs have been documented as powerful angiogenic inducers, combined heating and tensile stress conditioning may be a promising stress protocol that influences angiogenesis without exogenous delivery of GFs. Our study supports this concept by demonstrating that combined thermal and mechanical stress can increase VEGF secretion. Therefore, given that angiogenesis and VEGF are critical in the bone healing process [64], our stress conditioning protocols utilizing heating and cyclic tension may enhance VEGF-mediated communication between osteoblasts and endothelial cells. These protocols could potentially be used to stimulate blood vessel formation in a bone microenvironment or within bone scaffolds.

In conclusion, our study revealed that combined stress conditioning for short periods has the potential to modify cellular activity. Combined stress induced HSPs, upregulated OPN and OPG mRNA expression, increased VEGF secretion, and suppressed MMP-9 mRNA and secretion. Compared to heating or tension alone—which only affected some proteins—the combined stress treatments were able to produce changes across all proteins and enzymes investigated in this study. Therefore, our conditioning protocol of combined tensile stress (i.e., equibiaxial 3%, 0.2 Hz, intermittent mode of 10-second tension and 10-second rest) and thermal stress at 44°C for shorter duration than 10 minutes has the potential to impact bone healing and regeneration by upregulating cytoprotective proteins, modifying expression of bone-related proteins, and inducing synthesis of proteins essential for angiogenesis. Future research should focus on 3D* in vitro* studies that apply our combined thermal and mechanical stress protocol to cell-seeded constructs in order to develop bone tissue replacements suitable for healing bone defects. These experiments should investigate long-term conditioning using single or repeated stress treatments to investigate whether this could more effectively enhance bone ECM maturation. Overall, the ability of combined stress to broadly influence cellular protein production, without inducing apoptosis, is a beneficial strategy for bone tissue engineering.
